# Dynamic Control of Quantum Dot Localization in Nematic Liquid Crystal Matrix by Means of Photoinduced Phase Transition

**DOI:** 10.3390/molecules31010131

**Published:** 2025-12-30

**Authors:** Yaroslav Derikov, Alexander Ezhov, Oleg Karpov, Georgiy Shandryuk, Yuri Egorov, Olga Sokolovskaya, Leonid Golovan, Alexey Merekalov, Raisa Talroze

**Affiliations:** 1 A.V. Topchiev Institute of Petrochemical Synthesis, Russian Academy of Science, Moscow 119991, Russia; derikoff@yandex.ru (Y.D.); alexander-ezhov@yandex.ru (A.E.); o-karpov777@mail.ru (O.K.); shandgo@mail.ru (G.S.); yuegor@googlemail.com (Y.E.); 2 Faculty of Physics, Lomonosov Moscow State University, Moscow 119991, Russia; oi.sokolovskaja@physics.msu.ru

**Keywords:** quantum dots, nematic liquid crystal, azo-chromophores, light-induced phase transition, photoluminescence, microscopy, imaging

## Abstract

The stimulated assembly/disassembly of particles is a technique allowing for precise spatial and temporal control over the resulting structures to be realized. The application of a photosensitive liquid crystal (LC) allows the use of a photo-initiated order–disorder transition for the ordering and redistribution of dispersed nanoparticles. The semiconductor quantum dots (QDs) among them are useful for the imaging of such redistribution through simple luminescent microscopy with excitation by laser radiation at a wavelength of 532 nm. Doping the LC matrix with azo-chromophore molecules allowed us to localize the light-driven phase transition of the LC from the organized to the isotropic phase inside the spot, illuminated by ultraviolet (UV) light through a slit. The phase transition leads to a redistribution of the QDs within the matrix, followed by QD-rich region formation. After the termination of UV illumination, the QDs were found to form droplets in the region where UV illumination resulted in a homogeneous distribution of the QDs. The translation of the sample through the UV-illuminated spot resulted in QD accumulation inside the isotropic phase at the borders of the isotropic phase. The results obtained provide a good agreement with the model calculations of nanoparticle diffusion at the LC phase–isotropic liquid interface.

## 1. Introduction

Ensembles of semiconductor quantum dots (QDs), due to their unique properties, are extremely promising as a laser medium [1]. However, since for QD composite media the laser generation threshold strongly depends on the QD spatial distribution, tailoring their positions, e.g., the formation of QD-containing waveguides, gratings, or other structures, is of great importance. To achieve this goal, the QD can be arranged in a medium that allows a restructuring induced by external actions, e.g., in liquid crystal (LC). Among the widely studied materials, LCs have attracted much attention due to their unique physical properties such as orientational order, birefringence, high molecular mobility, etc. This aggregate state of matter, inherent in specific organic compounds of anisotropic form, is in a phase equilibrium between the crystalline state of a solid and isotropic liquids. The ability to orient and reorient LC molecules under external influence plays a key role in their use in various applications such as LC displays, optical lenses, infrared reflectors, and various photonic devices. Currently, the introduction of various inorganic particles into nematic liquid crystal is one of the most well-known and studied methodologies, since it improves the properties of nematic systems [2,3,4,5,6,7,8,9,10,11,12]. The LC matrix can drive the organization of introduced nanoparticles and provide broad opportunities to use such combined systems in quantum computing devices, photovoltaic devices, light-emitting diodes (LEDs), and many others, but various nanoparticles can also control the properties of the LC medium. There have been numerous attempts to improve the conductivity, anisotropy, and other characteristics of LCs by mixing them with inorganic QDs. However, few studies have examined the effect of changing the structure of QD surface ligands, which can significantly influence the coupling interaction with the LC environment and the final properties of the composite system [13,14], in particular, the photoinduced phase transition.

Since quantum dots themselves tend to aggregate, the choice of a QD stabilizing ligand plays a key role in the QD–medium interaction. For LC systems, the process of QD aggregation during the isotropic liquid–LC transition has been well studied and is associated with the thermodynamically driven tendency of the LC system to exclude sources of defects from its volume [15]. The introduction of mesogenic stabilizers into the QD shell is a common method for improving the compatibility of QDs with the LC matrix [13,14,15,16]. Such stabilizers also promote QD ordering during the isotropic liquid–LC transition. Mesogenic stabilizers in most cases contain aromatic rings, which are responsible for short-range π–π interactions between the shells of closely packed QDs, stabilizing the resulting structures [16].

The pivotal idea for controllable QD adjustment is the variation in the QD solubility, distribution, and concentration when the LC matrix undergoes a phase transition from the organized to the isotropic phase [10,11]. Controllable phase transitions allowing the desired structure to be formed can be performed with the help of photochromic additives in the LC molecule. The isotropization of the LC matrix caused by the photoinduced isomerization of the photochromic molecule, which takes place at a temperature below the isotropization temperature, causes a redistribution of the QD, which is a base for the production of a desired QD structure.

Previously, we reported the synthesis of CdSe/ZnS QD-LC composites based on cyanobiphenyl LC with embedded azobenzene-containing molecules and reported their applicability and demonstrated ‘order–disorder’ phase transition in them [12]. However, the prospective employment of these composite media for the formation of the QD structure needs a detailed knowledge of the structural, electronic, and recombinational properties of the QDs inside the QD-LC composite. In particular, we should obtain knowledge of the distribution and concentration of the QDs in the composite and their variations caused by light action, the possible sizes of the occurring QD structures and their temporal characteristics, the possibility to accumulate the QDs, etc. To reach these aims, we employed a photoluminescent (PL) microscopy technique for the detection of the rearrangement of CdSe/ZnS QDs in a matrix of nematic LC with embedded azo-chromophore molecules induced by UV illumination.

The main idea of the work could be demonstrated by the diagram shown in the graphical abstract. QD-rich microphases, or droplets, are uniformly distributed in the photosensitive LC matrix. Localized UV irradiation leads to an LC-isotropic phase transition in the exposed area, followed by QD homogenization in the isotropic melt. Moving the sample relative to the UV-irradiated spot could lead to the accumulation of QDs inside the isotropic phase area.

## 2. Results and Discussion

In our study, we used an LC-QD composite based on nematic LC with embedded 10 wt.% azo-chromophore molecules undergoing a configurational (E-Z) transition stimulated by UV light and containing 0.1 wt.% CdSe/ZnS QDs. The composite is characterized by an isotropization temperature ($T_{NI}$) of 60 °C, as was previously found with the help of a differential scanning calorimetry technique and dielectric permittivity measurements [4].

### 2.1. PL Spectrum

**Figure 1:**
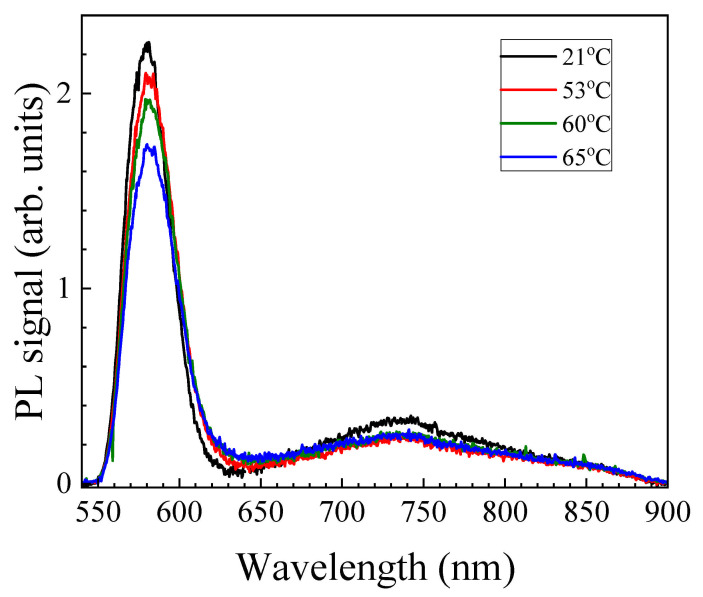
PL spectra of LC-QD composites at different temperatures.

The employed sample of the QD-LC composite demonstrates an easily seen PL under excitation from laser radiation at 532 nm (Figure 1). The PL spectrum of the sample includes two bands with maxima at 580 and 725 nm, which can be attributed as exciton and defect ones; the latter band is associated with defects on the surfaces of QDs, e.g., selenium atom vacancies acting as charge carrier traps [17]. The variation in the sample temperature does not change the form of the PL spectrum, although the PL intensity slightly decreases with the temperature increase. It is worth noting that a rise in the temperature above *T_NI_* results in a noticeable fall of the exciton band signal, whereas the defect band remains stable.

### 2.2. Dynamics of the QD Distribution at a Fixed Position

All the results mentioned below on the dynamics of the QD redistribution in the LC matrix were obtained at a temperature of 53 °C, i.e., 7 °C below *T_NI_*.

**Figure 2:**
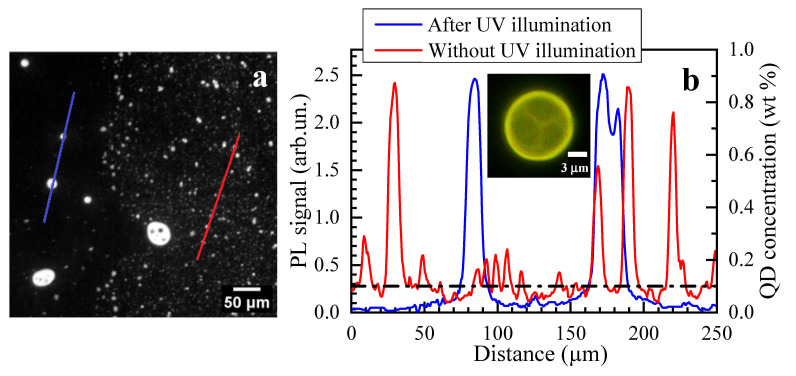
PL microscopy image of the sample region partially illuminated by UV light (left part of the image) (**a**) and PL and QD concentration distributions measured along the highlighted cross-sections in the region exposed to UV radiation (left cross-section, blue line) and in the region not exposed to UV radiation (right cross-section, red line) (**b**). QD concentration is recalculated from PL intensity. Dash–dot line corresponds to average initial distribution equal to 0.1 wt.%. Inset: the PL image of a QD-rich droplet.

Details of the QD distribution in the composite QD-LC medium can be found from the PL microscopy images shown in Figure 2a. The initial (unilluminated) sample region demonstrates both a rather dim inhomogeneous background and much brighter droplets of about 10 μm in diameter (Figure 2b) enriched with QDs. As one can see, the inhomogeneity of the PL signal, and therefore the QD concentration, reaches up to 1:20 contrast. Converting the PL intensity to the QD concentration, we can conclude that the concentration of QDs in the QD-rich regions is 0.9 wt.% (for a mean concentration of 0.1 wt.% [3]), while in LC regions this value is 0.05 wt.%.

**Figure 3:**
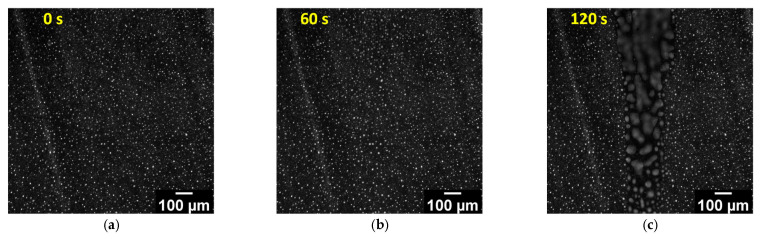

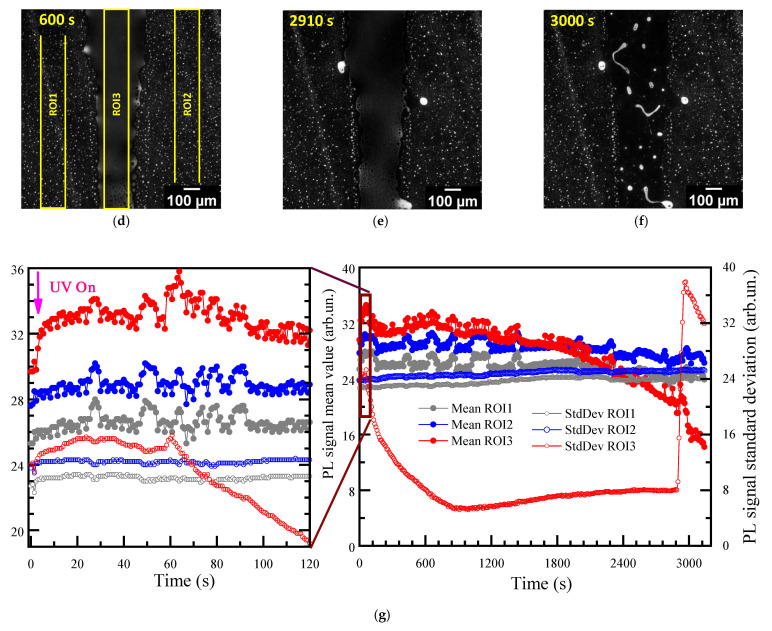
Time evolution of the QD emission distribution during the local UV illumination in a stripe of 150 µm before start of the illumination (**a**), after illumination for 60 s (**b**), 120 s (**c**), and 600 s (**d**), and 20 s (**e**) and 110 s (**f**) after finishing the UV illumination; (**g**) dependence of the PL signal mean values and the PL signal standard deviations in different ROIs (shown in panel (**d**)) on time; period of the first 120 s is shown separately on the left panel.

Figure 3a–f present PL microscopy images of the sample before, during, and after its UV illumination. The temporal dependences of the PL signals integrated in different regions of interest (ROIs), which are stripes of 150 µm in width (Figure 3d), are shown in Figure 3g. As one can see, initially, mean PL signals for different ROIs differ noticeably, although the difference is less than 12%; this effect is determined by variations in the initial QD concentration in the sample studied. Moreover, before the illumination starts, the PL signal varies significantly for different small regions, which is determined by variations in the initial spatial QD concentration in the studied sample caused by the redistribution of QDs between the LC phase and droplets enriched by QDs. This redistribution is induced by the transition from the isotropic to the LC phase of the matrix at a temperature lower than *T_NI_*.

For unilluminated regions, neither the images nor the PL signals integrated over the ROIs significantly vary with time. In contrast, illuminated region (ROI3) is characterized by a discernible redistribution of the emitting QDs. At the start of the UV illumination, the PL signal integrated over ROI3 increases up to 10%, which is a result of additional PLs excited by UV light. As one can see from Figure 3b,g, the very first variation in the PL microscopy image was found after UV illumination for 1 min, which causes an additional 10% PL signal increase. Illumination over a longer time results in the homogenization of the QD distribution inside the stripe of about 200 μm in thickness, which slightly exceeds the UV illuminated region, caused by the isotropization of the LC matrix inside the stripe. After UV illumination termination, QD-enriched droplets with a higher diameter than the ones in the initial LC-QD composite are formed (Figure 2b). They are surrounded by regions depleted in QDs; initial microphases with a moderate QD content could be hardly found after the UV exposure. The contrast of the PL signals increased in comparison with the initial one and reached up to 1:40, with a QD concentration less than 0.02 wt.% outside the droplets (Figure 2b).

It is worth noting that the standard deviation of the PL intensity calculated in the frames of the whole ROI can serve as a very useful quantitative characteristic of QD concentration equalization during the isotropization of the composite matrix caused by UV illumination. In the UV-illuminated region (ROI3), the standard deviation value starts to fall drastically after 1 min of illumination, which is in precise agreement with the variation in the PL microscopy image (Figure 3b and enlarged fragment of Figure 3g). A complete homogeneity of the QD distribution, which corresponds to a decrease in the standard deviation more than three times due to the homogeneous QD distribution in the isotropic phase, is achieved within 10 min of UV irradiation (Figure 3d,g). Note, however, that during this period mean value of the PL signal has no significant variations. The slow fall of the mean PL signal taking place after UV illumination for 15 min is possibly connected with the QD degradation under continuous UV illumination. The formation of the QD-enriched droplets after UV illumination termination results in a huge rise in the PL signal standard deviation (Figure 3g for time exceeded 2900 s). Thus, the formation of QD-enriched droplets upon switching on and off UV irradiation is reversible at temperatures slightly lower than $T_{NI}$.

The maximum speed of movement of the region exposed to UV radiation will be determined by the time it takes for the composite to achieve a uniform concentration of QDs. After the composite matrix becomes isotropic under UV radiation, a leveling diffusion of quantum dots from the regions enriched with them must occur.

Fick’s second law predicts how diffusion causes concentration to change with respect to time. It is a partial differential equation which in one dimension reads:

(1) $$\frac{\partial C}{\partial t}=D\frac{\partial^{2}C}{\partial x^{2}},$$

where C is the *QD* concentration, t is time, D is the diffusion coefficient, and x is the position. The solution to Equation (1) on an infinite interval is the convolution of the initial concentration data and the kernel:

(2) $$C\left(x,t\right)=\frac{1}{\sqrt{4\pi Dt}}\;\exp\left(-\frac{x^{2}}{4Dt}\right).$$

The diffusion coefficient for a single *QD* may be estimated using the Stokes–Einstein–Sutherland equation for the diffusion of spherical particles through a liquid with a low Reynolds number:

(3) $$D=\frac{k_{B}T}{6\pi \eta r_{QD}},$$

where $k_{B}$ is the Boltzmann constant, T=326 K is the absolute temperature, η is the dynamic viscosity of the matrix in the isotropic phase, and $r_{QD}$ is the Stokes radius (effective solvated radius in solution) [18] of the spherical QD.

**Figure 4:**
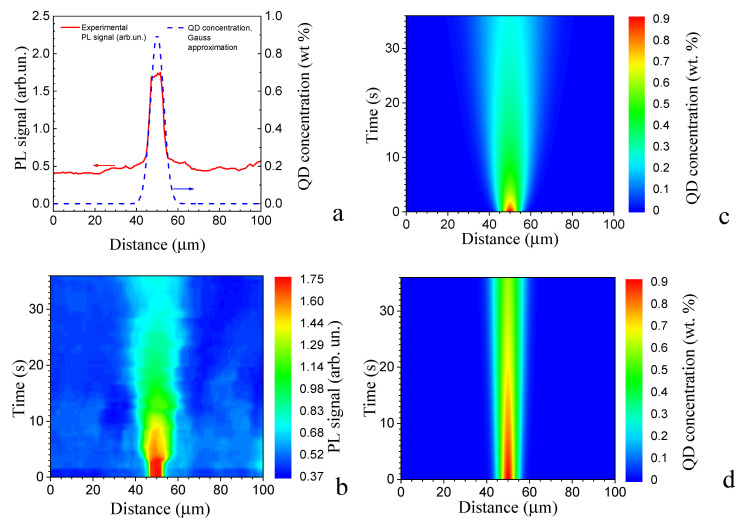
Initial PL intensity profile and its Gauss approximation (**a**), time dependence of the PL profile on the time elapsed after the onset of UV irradiation (**b**), and simulated dependences obtained for two different values of the product of the dynamic viscosity and the Stokes radius of the QD: $\eta r_{QD}=$ 1.25 × 10^−10^ N s/m (**c**) and $\eta r_{QD}=$1.25 × 10^−9^ N s/m (**d**).

Since we have no precise information on either the dynamic viscosity of our matrix or the Stokes radius of the QD, the product of the dynamic viscosity and the Stokes radius of the QD $\eta r_{QD}$ was evaluated to estimate the diffusion coefficient. The radius of the QD determined from the TEM results is 2.5 nm. For nematic LCs not far from $T_{NI}$, the mean dynamic viscosity may be estimated by an order of magnitude as 0.05 Pa·s [19]. Thus, the product $\eta r_{QD}$ can be estimated as 1.25 × 10^−10^ N·s/m. On the basis of the obtained estimate, we simulated the leveling diffusion from the QD-enriched droplet and estimated the typical time it takes. For this purpose, a single droplet enriched with the QDs 7 µm in diameter was selected, with the initial profile shown in Figure 4a. Figure 4b presents the experimentally found dependence of its PL profile on the time elapsed after the start of UV irradiation, which demonstrates the significant fall of the QD concentration in the time interval of 35 s. Variations in both the size of the QD-enriched droplets and the QD concentration in it were estimated through the direct solution of Equation (1) for the cases of the initial QD concentration profile approximated by the Gauss function, with a maximum of 0.9 wt.% (Figure 2b), and two different values of the product $\eta r_{QD}$: 1.25 × 10^−10^ N s/m (Figure 4c) and 1.25 × 10^−9^ N s/m (Figure 4d). As one can see, for the former value of the product $\eta r_{QD}$ and the higher diffusion coefficient, the PL signal decreases almost 4 times, whereas the size of the droplet increases more than twice for 35 s, i.e., the QD-enriched droplet is re-dispersed in less than 1 min (Figure 4c). Estimations for the higher value of the product $\eta r_{QD}$ and lower diffusion coefficient demonstrate a significantly slower dissolving of the droplet, exceeding the experimentally found one (Figure 4d).

Thus, comparing experimental and simulation results, we can find a good qualitative agreement of the simulation for viscosities of 0.05 Pa·s (Figure 4c) and the data from PL microscopy (Figure 4b). The simulation gives us the estimation of the QD diffusion rate, which is about 40 µm/min by the value order.

### 2.3. Dynamic Accumulation of the QDs with the Help of Moving UV Light Spot

**Figure 5:**
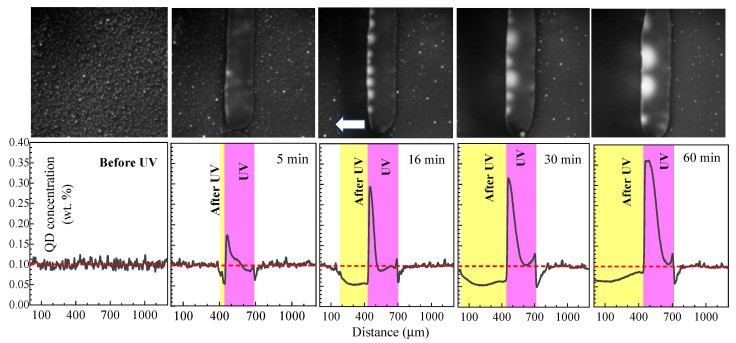
PL microscopy images (**upper line**) and averaged over 500 µm in the direction across the travel cross-sections of QD concentration distributions (**bottom line**) in the moving (**right** to **left**) sample continuously illuminated for 0, 5, 16, 30, and 60 min by UV light through the mask spot. The dashed line corresponds to the initial QD concentration (0.1 wt.%). The sample movement speed is 26 µm/min. Direction of the sample movement is shown by arrow.

The movement of the UV-illuminated spot over the sample allows the QDs to be accumulated at the edges of the spot. The important parameter is the speed of the light spot movement, which should not exceed a certain limit for a steady rise in the PL signal with the light spot movement. According to previous results, the speed should be 10 µm/min in order of magnitude, whereas a higher speed cannot result in a complete QD redistribution. Figure 5 presents PL microscopy images and, averaged over 500 µm in the direction across the travel line cross-sections, the QD concentrations for various illumination times and positions of the illuminated field at the sample surface for the sample moving right to left with a speed of 26 µm/min. For 5 min of illumination, based on our previous observation of the disappearance of the QD droplets in the isotropic phase of LC, we can attribute the obviously distinguished region in the center of the images as an isotropic phase surrounded with an organized phase containing the QD-rich droplets. The width of the isotropic phase region is about 250 μm, which exceeds the mask slit width (170 μm) and slightly increases with time. For longer illuminations, returning the LC from the isotropic to the nematic phase results in the QDs’ exclusion from the amorphous region, as was predicted in Ref. [10]. Thus, the depletion in the QDs takes place in the previously illuminated region (left to the isotropic phase spot); the QD concentration in this region falls up to two times, combined with an increase in the QD concentration more than three times in comparison with the unilluminated region at the back (left) edge of the isotropic spot. However, for the UV illumination of 1 h, a small increase in the QD concentration in the depleted region is found (cf. PL signal cross-sections for 30 and 60 min).

**Figure 6:**
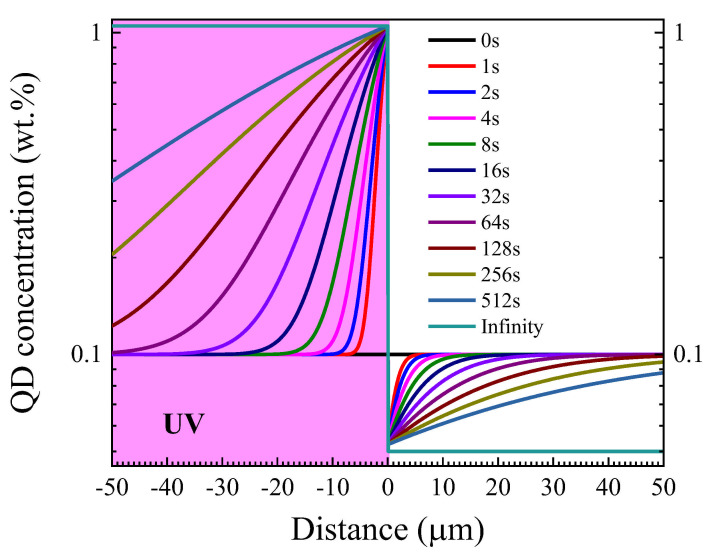
Time dependence of QD concentration distribution at the interface between the isotropic (negative x) and LC (positive x) phases.

A characteristic feature of the QD concentration distribution is a strong inhomogeneity near the interface between the isotropic and LC phases. Near this interface, the LC phase is depleted in QDs, while the isotropic phase is enriched in them, with the QD concentration near the interface being higher than in the isotropic phase located far from the interface. This feature is explained by the finite lifetime of the interface between the isotropic and LC phases. To confirm this statement, a simulation of the dependence of the QD concentration near the interface was performed. The product $\eta r_{QD}$ was equal to 1.25 × 10^−10^ N s/m, and the equilibrium concentrations of QDs were 0.1 wt.% for the initial LC phase and 1.05 wt.% for maximum in the isotropic phase. The simulation results are shown in Figure 6. These results demonstrate a noticeable inhomogeneity of the QD concentration and are consistent with the experimental results. Indeed, the simulation results for the right (front) edge of the UV illuminated region, where the isotropic liquid meets the organized phase, qualitatively describe abrupt jumps of the QD concentration as well as an increase in the thickness of both QD-enriched and QD-depleted zones with illumination time. Generally, the asymmetry of the QD distributions, i.e., the accumulation of a greater amount of the QDs at the left (back) edge of the illuminated region, is a result of the sample movement from right to left. The QDs moving with the sample are unable to penetrate the phase interface. However, this consideration is valid for the low speed of the sample, which does not exceed the diffusion speed.

**Figure 7:**
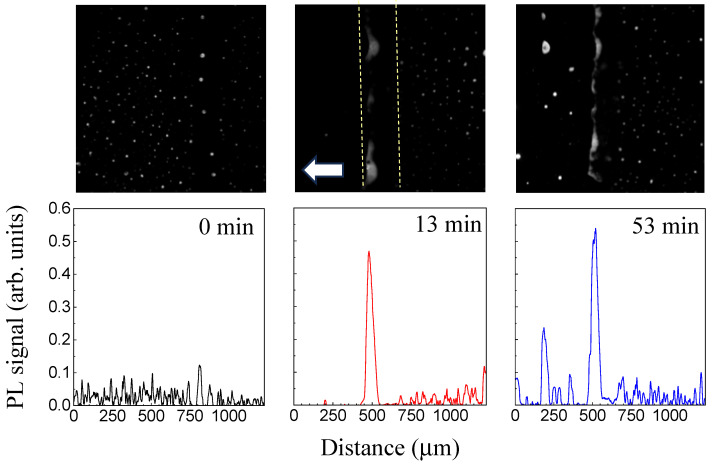
PL microscopy images (**upper line**) and profiles (**bottom line**) of the PL signal for the sample moving from right to left, continuously illuminated for 0, 13, and 53 min. The sample movement speed is 87 µm/min. Direction of the sample movement is shown by arrow. The UV-illuminated region is shown by dashed lines.

In contrast, the higher movement speed (87 µm/min), significantly exceeding the diffusion speed, results in a totally different QD distribution. The UV-illuminated region also demonstrates a depletion in the QD concentration for an illumination for 13 min; however, further illumination results in the detachment of rather large droplets (up to 100 µm) enriched with the QDs (Figure 7). It is noticeable that, in contrast to the case of lower speed for the sample movement (Figure 5), the QDs are partially accumulated at the left (back) edge of the amorphous spot and partially overcome the barrier caused by the QD diffusion and are deposited as QD-enriched droplets in the QD-depleted region behind the left (back) edge, with the step-like increase in QD concentrations at the front edge of the isotropic spot being not formed. These facts indicate that the sample speed of 87 µm/min is higher than the speed of the QD diffusion. Estimating UV exposure times yields values of 350 s and 100 s for the velocities 26 µm/min and 87 µm/min, respectively. A comparison of the obtained UV exposure values with estimates based on static measurements demonstrates their consistency.

## 3. Materials and Methods

We employed a eutectic mixture of cianobiphenyl-based low-molecular-weight mesogenic molecules as a nematic LC matrix; the compound forms only a nematic phase in the temperature range of −30 to +64 °C [12]. To ensure the control of transitions between anisotropic and isotropic phases, the photochromic azo-compound AX-8 was introduced into the LC mixture (10 wt.%). Spherical CdSe/ZnS QDs of an average diameter of about 5 nm, stabilized with mesogenic carboxyl acid, were used in the mixture, with a fraction of 0.1 wt.%. The QD surface stabilizer was used to improve QD solubility. The isotropization temperature variation after the QD introduction is less than 1 °C, which indicates no significant effect of the QDs at the used concentration on LC ordering. Detailed information on the synthesis of the compound is contained in Appendix A.

**Figure 8:**
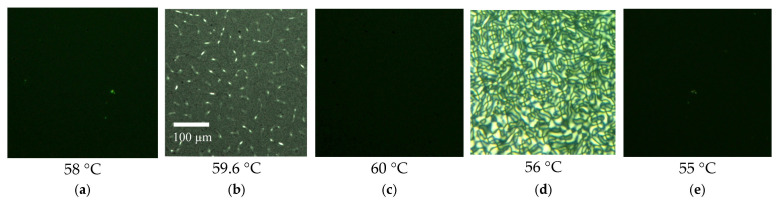
POM images of the LC-QD composite placed between two glass slides for different temperatures during heating (**a**–**c**) from 58 to 60 °C and cooling (**d**,**e**) back to 55 °C.

The obtained mixtures were placed between two glass slides divided by a spacer of 25 µm thickness. The glass slides were rinsed with ethanol and wiped dry before use. The chosen cell depth is optimal to provide a highly detectable PL signal and to avoid redistribution of the QDs in depth. The mixture demonstrated properties of homeotropic LC for temperatures below $T_{NI}$ = 60 °C. Polarizing optical microscopy (POM) in crossed polarizers demonstrated a black field for temperatures below 59.6 °C (Figure 8a), which is typical for homeotropic media, a schlieren texture at 59.6 °C (Figure 8b), which accompanies the reorientation of mesogens during the process of transition from anisotropic to isotropic phases, and a black field again for the isotropic phase of the LC at a temperature of 60 °C (Figure 8c); cooling of the sample results in the occurrence of a more pronounced schlieren texture at 56 °C (Figure 8d), which is also a manifestation of the phase transition into the LC phase, and a black field for the homeotropic LC phase at a lower temperature (Figure 8e).

PL microscopy is the main experimental technique employed to study spatial redistributions of the QDs. The measurements were carried out with the help of the optical microscope POLAM L-213 (LOMO, St. Petersburg, Russia), equipped with a thermal stage FP82HT (Mettler Toledo, Greifensee, Switzerland). The temperature of the tested samples was 53 °C, which is 7 °C below the isotropization temperature [4], and maintained with an accuracy of 0.1 °C. The standard halogen light source of POLAM L-213 has been replaced by a high power 365 nm LED CUN6GF1A (Seoul Viosys, Ansan, Republic of Korea) and a Wood optical filter. For the PL excitation radiation, a solid-state laser (OXlasers, Shanghai, China) at wavelength of 532 nm was used, which does not cause any configuration change in the azo-chromofore. The illumination of the sample is homogeneous (Supplementary Materials, Figure S1). The light from the laser was delivered to the thermal stage by an optical fiber equipped with a pair of collimators. Light intensities at the sample surface were up to 0. 01 W/cm^2^ for UV radiation and 0.025 W/cm^2^ for radiation at a wavelength of 532 nm, correspondingly. A notch filter and absorption optical filter separated the PL signal before reaching the camera, based on a CMOS sensor IMX269 (Sony, Tokyo, Japan). PL spectra of the studied QD-LC composites were obtained through excitation with pulse radiation from a laser PL2143A (Ekspla, Vilnius, Lithuania) at wavelengths of 532 nm (25 ps, 10 Hz, fluence 2 mJ/cm^2^) and registered with the help of a spectrometer Spectra Pro 2500i (Princeton Instruments, Acton, MA, USA). The obtained images and videos were processed using the software ImageJ version 1.54 g (National Institutes of Health, Bethesda, MD, USA). To minimize the impact of UV radiation and 532 nm laser radiation on the processed images, the camera’s red channel was used for measurements. The mean PL value and its standard deviation (StdDev) were chosen as characteristics describing the evolution of QD PL upon exposure of the composite to UV radiation. The regions of interest (ROIs) used for measuring are 150 µm by 1200 µm in dimension. Each ROI, consisting of approximately 450,000 pixels, was used to calculate mean PL and StdDev values. StdDev in this case characterized the presence of heterogeneity in the QD distribution and significantly decreased under UV irradiation. The contribution of the noise signal to StdDev was significantly smaller than the contribution from the spatial heterogeneity of the QD distribution.

A steel diaphragm with a cut 170 μm wide and a 1.8 mm long slit was placed onto the heating stage to shape the UV spot for localized illumination. For the static experiments, the UV light was passed through the diaphragm, forming a stripe crossing the entire observable sample area with homogeneous illumination. The slit width was found to be optimal for the dynamic experiment, since it allows us to detect separated QD-enriched regions at both borders of the light-illuminated region. In dynamic experiments, the sample slide moved at constant speeds of 26 or 87 μm/min, provided by a 1 rpm electric motor attached to the positioner wheel of the thermal stage through a gearbox, and was continuously UV-illuminated through the immobile slit.

## 4. Conclusions

Thus, we have demonstrated the redistribution of CdSe/ZnS QDs under the influence of UV radiation in composites based on the nematic LC containing the azo-chromophore. UV irradiation leads to the transition of the LC from the nematic to the isotropic phase, followed by the homogenization of the QD distribution. This homogenization is accompanied by the gradual disappearance of QD-enriched droplets, with diameters ranging from a few microns to 10 μm. The reverse process, with the appearance of QD-enriched droplets, is observed upon cessation of UV irradiation. It is shown that the characteristic times for the homogenization of the QD distribution in a matrix subjected to UV irradiation are 10–15 min for regions with characteristic sizes on the order of 100 μm. The concentration of the QDs can be significantly increased by moving the UV-illuminated spot relative to the sample at a velocity not exceeding the quantum dot diffusion rate. The translation of the UV-illuminated spot through the sample results in QD accumulation inside the isotropic phase induced by UV irradiation.

## Figures and Tables

**Figure A1:**
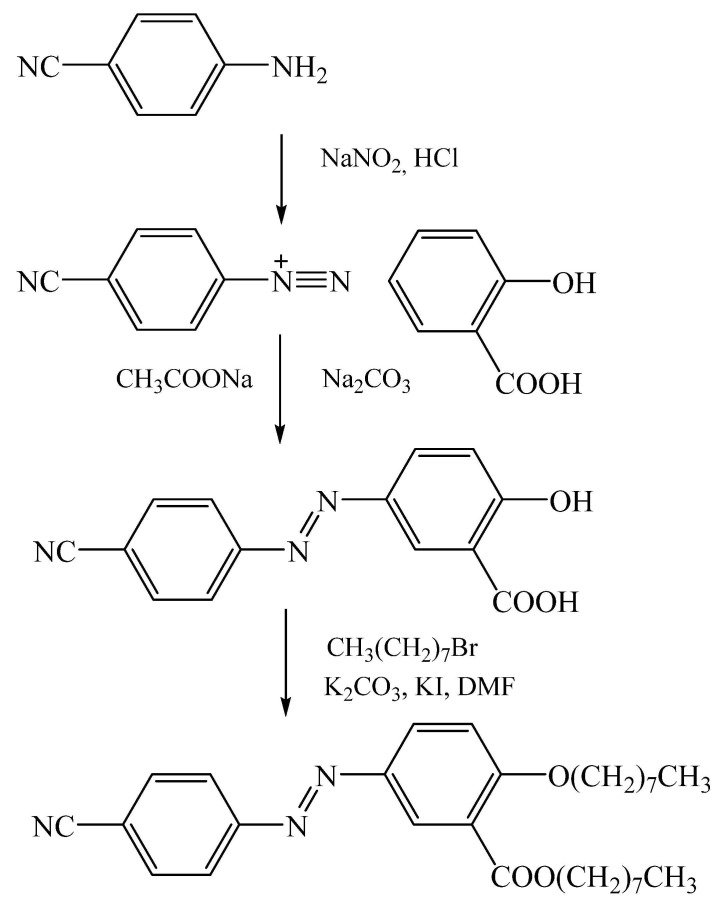
Synthesis of the photochromic azo compound AX-8.

## Data Availability

The data underlying this article are available in the article and Supplementary Materials. Video of dynamics of the QD distribution in LC matrix caused by UV illumination at a fixed position can be found at https://vkvideo.ru/video-155741472_456239017 (accessed on 23 December 2025).

## Supplementary Materials

The following supporting information can be downloaded at https://www.mdpi.com/article/10.3390/molecules31010131/s1. Text S1: NMR data; Figure S1: Illumination of the sample with laser radiation at wavelength of 532 nm; Figure S2: Dependence of the PL signal on the QD concentration in the LC-QD compound.

## Abbreviations

The following abbreviations are used in this manuscript:

PL	Photoluminescence
LC	Liquid crystals
POM	Polarizing optical microscopy
QD	Quantum dots

## Appendix A. Synthesis of the LC-QD Compound

### Appendix A.1. Synthesis of CdSe/ZnS QDs

For the synthesis of QDs, two solutions were prepared. To obtain the first solution, 0.2 mmol of cadmium oxide, 2 mmol of zinc acetate, and 9 mmol of oleic acid were mixed in 10 mL of 1-octadecene. The reaction mixture was then evacuated and purged with argon to create an inert atmosphere. It was gradually heated to 150 °C under an argon flow and maintained for one hour until the complete dissolution of cadmium oxide and zinc acetate, resulting in the formation of cadmium and zinc oleates in the reaction mixture. The first solution was then heated to 280 °C, and the second solution, containing 0.2 mmol of selenium and 2 mmol of sulfur dissolved in 1.5 mL of trioctylphosphine, was swiftly injected into it. Upon mixing these two solutions, the process of CdSe/ZnS QD formation commenced. The reaction was carried out for 6 min and then rapidly cooled to room temperature to stop nanoparticle growth. Purification was carried out via triple precipitation in acetone (centrifuging for 10–15 min at 6000 rpm), redissolving the precipitate in toluene if necessary.

As a result, spherical CdSe/ZnS QDs with an average diameter of about 5 nm were obtained, stabilized by oleic acid and trioctylphosphine as organic ligands. The formed QDs are insoluble in both the isotropic and the LC phases of the matrix. To render the obtained nanoparticles soluble in the isotropic phase of a nematic liquid crystal, their surface was further modified by replacing oleic acid with 2-(4″-Hexyl-2′-chloro-[1,1′:4′,1″-terphenyl]-4-yloxy)propanoic acid; its synthesis was described in detail elsewhere [20].

The choice of this substance as a new stabilizer is due to the presence of a functional carboxyl group, which allows it to interact with the cations on the QD surface directly and replace oleic acid and improve of the QDs’ affinity for the LC matrix, which ensures a uniform distribution of nanoparticles throughout the volume in the isotropic phase. The ligand exchange reaction was carried out with an excess of the new stabilizer. For this purpose, 0.05 g of 2-(4″-Hexyl-2′-chloro-[1,1′:4′,1″-terphenyl]-4-yloxy)propanoic acid was dissolved in 0.2 mL of tetrahydrofuran (THF) and mixed with 0.5 mL of a CdSe/ZnS QD sol in THF (concentration 0.02 g/mL). The reaction mixture was kept for three days under an inert atmosphere. Purification was performed by precipitation in acetone and centrifugation (6000 rpm for 10 min).

### Appendix A.2. Synthesis of the Photochromic Azo Compound AX-8

The synthesis of the photochromic azo compound AX-8 with C8 alkyl radicals in the aromatic ring was performed in two stages via the diazotization of 4-aminobenzonitrile, followed by the reaction of the resulting 5-(4-cyanophenylazo)-2-hydroxybenzoic acid with 1-bromooctane, yielding the target compound—octyl 5-(4-cyanophenylazo)-2-octyloxybenzoate (Figure A1).

The following reagents and solvents were used for the synthesis without further purification: 4-aminobenzonitrile (Acros Organics, 98%), 1-bromooctane (Acros Organics, 99%), 2-hydroxybenzoic acid (Sigma-Aldrich, ACS reagent grade, >99%), N,N-dimethylformamide (Acros Organics, 99%), ethanol (Komponent-reaktiv, 96%), sodium acetate (Komponent-reaktiv, analytical grade), anhydrous sodium carbonate (Alfa Aesar, 99%), potassium iodide (Fluka, 99%), potassium carbonate (Komponent-reaktiv, analytical grade), sodium nitrite (Fluka, 99%), and 36% hydrochloric acid (reagent grade, 35–38% HCl).

5-(4-Cyanophenylazo)-2-hydroxybenzoic acid was synthesized through the diazotization of 17.7 g (0.15 mol) of 4-aminobenzonitrile in a 15% (*w*/*w*) HCl solution, followed by coupling of the diazonium salt with 20.7 g (0.15 mol) of 2-hydroxybenzoic acid in a sodium carbonate solution according to the well-known procedure [21]. After completion of the reaction and neutralization of the mixture, 37.2 g of the crude product was isolated. The yield of the pure product after recrystallization from ethanol was 35.6 g (90%).

To a solution of 8.01 g (0.03 mol) of 5-(4-cyanophenylazo)-2-hydroxybenzoic acid in 25 mL of dry DMF, 4.5 g of potassium carbonate and 0.5 g of potassium iodide were added. The solution was heated to 90 °C with stirring and maintained for 40 min. Then, 10.5 mL (0.06 mol) of 1-bromooctane was added, the temperature of the reaction mixture was raised to 120 °C, and it was stirred for an additional 2 h. After cooling, the mixture was diluted with 250 mL of distilled water. The precipitated product was collected on a glass filter, washed extensively with distilled water, and dried in vacuo. Recrystallization from ethanol afforded 14.4 g (98%) of the pure product.

### Appendix A.3. Preparation of LC Matrix Mixtures with Azochromophore and Modified QDs

The nematic LC matrix used in this work consists of 4-pentyl-4′-cyanobiphenyl (48.5 wt.%), 4-propoxy-4′-cyanobiphenyl (18.2 wt.%), 4-amyloxy-4′-cyanobiphenyl (17 wt.%), trans-4-butylcyclohexanecarboxylic acid 4-ethoxyphenyl ester (10 wt.%), and 4-cyano-4′-diphenyl ether of 4-trans-butylcyclohexanecarboxylic acid (6.3 wt.%). Its clearing point is 64 °C.

As was previously shown [4], the optimal content of the azo-chromophore in the LC matrix for controlling its transitions under irradiation is 10 wt.%. The CN-AX8 azo-chromophore was chosen as the primary one. The process of creating mixtures of the LC matrix, azo-chromophore (10.0 wt.%), and QDs (0.1 wt.%) was carried out in two stages. First, all components were mixed in a THF solution and then dried to a constant weight. Subsequently, the mixture was heated to 100 °C and held until all components were completely dissolved in the matrix. The obtained mixtures were analyzed using a fluorescence microscope between two glass slides; the mixtures were also applied from the isotropic phase at 100 °C. During our preliminary experiments, we varied the QD concentration in the matrix from 0.1 to 1 wt.% and have found that for the QD mass fraction that occurred in this study’s integral PL signal for the sample is proportional to the QD mass fraction (Supplementary Materials, Figure S2).
