# Supplementary material for: Dynamic Control of Quantum Dot Localization in Nematic Liquid Crystal Matrix by Means of Photoinduced Phase Transition
Source: Molecules. 2025 Dec 30;31(1):131. doi: 10.3390/molecules31010131 (PMC12788157; doi:10.3390/molecules31010131)
Supplement: Supplementary file 1 [file molecules-31-00131-s001.zip › molecules-3991644-supplementary.pdf]

Supplementary materials:

NMR data

5-(4-Cyanophenylazo)-2-hydroxybenzoic acid

^{1}H-NMR: (DMSO-d_6, 400 MHz,): δ (ppm) 8.35 (d, J = 2.5 Hz, 1H), 8.08 (dd, J = 8.9, 2.6 Hz, 1H), 8.06 – 7.92 (m, 4H), 7.16 (d, J = 8.9 Hz, 1H).

Octyl 5-(4-cyanophenylazo)-2-octyloxybenzoate.

^{1}H-NMR ($CDCl_3$, 80 MHz): δ (ppm) 8.41 (d, J = 2.6 Hz, 1H), 8.24 – 7.60 (m, 5H), 7.08 (d, J = 8.8 Hz, 1H), 4.23 (dt, J = 16.6, 6.5 Hz, 4H), 1.78 (m, 4H), 1.32 (s, 20H), 0.88 (m, 6H).

Photoluminescence microscopy

1. The image of the entire observed region for the test sample of CdSe/ZnS QDs dispersed in 1-octagecene under excitation by laser radiation at wavelength of 532 nm is shown in Fig. S2.1. The distribution of laser radiation in the region could be described as homogeneous. The darkening at the corners of the image refers to the imperfections in the microscope optics, namely, lens vignetting and does not affect excitation homogeneity.

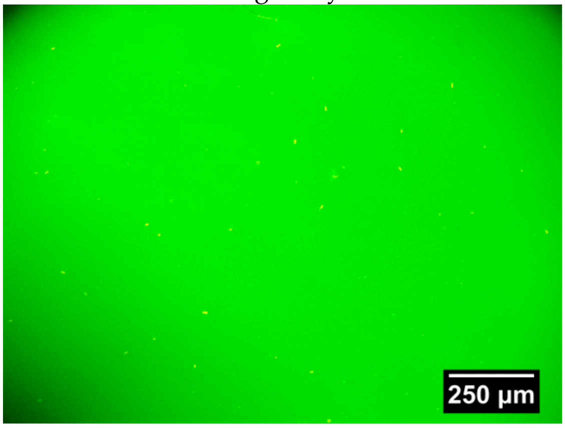

Figure S1. Illumination of the sample with laser radiation at wavelength of 532 nm.

2. Dependence of the PL signal on the QD concentration in the LC-QD compound obtained for the QD mass concentrations ranged from 0.1 to 1 wt. %. The PL signal was integrated over the central part (of 1x1mm) of the PL microscope image for at least three different areas of each sample. The QDs were homogeneously distributed in isotropic liquid at temperature above T_{NI}. For the QD concentrations below 1 wt. % the dependence is close to linear.

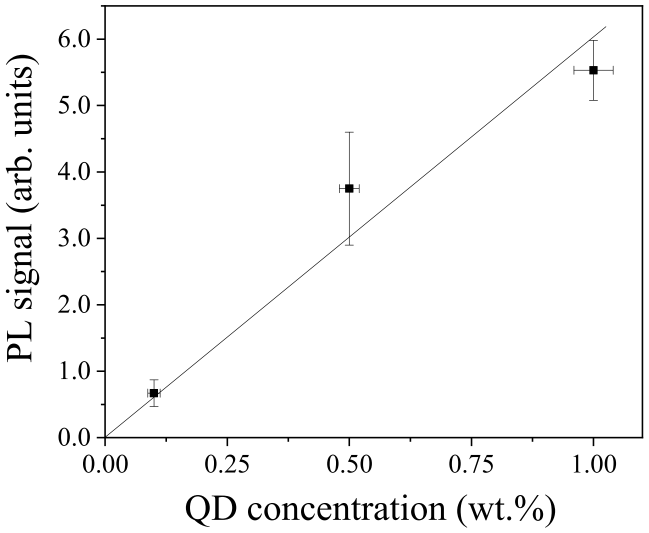

Figure S2. Dependence of the PL signal on the QD concentration in the LC-QD compound.
